# A Novel Immunochromatographic Strip for Antigen Detection of Avian Infectious Bronchitis Virus

**DOI:** 10.3390/ijms20092216

**Published:** 2019-05-06

**Authors:** I-Li Liu, Yi-Chun Lin, Yong-Chong Lin, Cai-Zhen Jian, Ivan-Chen Cheng, Hui-Wen Chen

**Affiliations:** 1 Institute of Veterinary Clinical Science, School of Veterinary Medicine, National Taiwan University, Taipei 10617, Taiwan; liuili@ntu.edu.tw; 2 Department of Veterinary Medicine, National Taiwan University, Taipei 10617, Taiwan; ycl2015@ntu.edu.tw (Y.-C.L.); stoneage0916@gmail.com (Y.-C.L.); r05643003@ntu.edu.tw (C.-Z.J.); ivancheng@ntu.edu.tw (I.-C.C.)

**Keywords:** immunochromatographic strip, infectious bronchitis virus, antigen detection, monoclonal antibodies, gold nanoparticles

## Abstract

Avian infectious bronchitis virus (IBV) causes considerable economic losses in the poultry industry worldwide, including Taiwan. IBV is among the most important pathogens in chickens, and it spreads rapidly among flocks. In addition to dozens of known serotypes, new viral variants have emerged due to the viral evolution and antigenic variation in IBVs. Therefore, the development of a sensitive, specific, and easily performed assay is crucial for the rapid detection and surveillance of IBV infections. A rapid and simple immunochromatographic strip (ICS) was developed in this study by employing monoclonal antibodies against spike and nucleocapsid proteins of IBV as the tracer and the capture antibody. The ICS showed high specificity in detecting IBV antigens, including several IBV genotypes and novel variants, as opposed to three other common avian respiratory viruses. The detection limit of the strip reached 10^4.4^ 50% embryo-infective dose. Moreover, in the experimental chicken model, the strip test demonstrated consistency in detecting IBV with RT-PCR gene detection. Taken together, this antigen detection strip has the potential to serve as an on-farm rapid test for IBV; therefore, it may facilitate surveillance and control of the disease.

## 1. Introduction

Avian infectious bronchitis virus (IBV) is a positive-sense single-stranded RNA virus belonging to the genus *Gammacoronavirus*, family *Coronaviridae*. IBV can infect the respiratory and urogenital systems in growing chickens, causing high mortality, slowing down growth, and reducing egg production in the flock [1]. Therefore, IBV may result in a considerable economic loss in the global poultry industry. In Taiwan, IBV first appeared in 1958, and the disease has occurred frequently, although an IBV vaccination program has long been implemented [2]. IBV strains in Taiwan include two genotypes, namely Taiwan Group I (TW-I) and Taiwan Group II (TW-II), which are prominent because of their capacity to cause nephritis and respiratory symptoms in chickens [3,4]. Because of its frequent gene mutations and recombination events, IBV variants in different serotypes have been isolated from the field constantly [5]. Due to serotype differences in Taiwan, extensive use of commercially available Massachusetts (Mass) serotype vaccines did not effectively prevent IBV outbreaks in chicken flocks [2].

Molecular studies reported that IBV contains four major structural proteins: The spike (S) glycoprotein, the phosphorylated nucleocapsid protein (N), the envelope protein (E) and the membrane glycoprotein (M). The S glycoprotein is believed to be related to the host range, and it can be post-translationally cleaved into S1 and S2 subunits [6]. The S1 protein is highly variable and is the primary target for neutralizing antibodies [7,8]. Moreover, the N protein is a relatively conserved structural protein that reveals high identity among various strains [9], and its highly immunogenic nature can induce antibodies as well as the T-cell mediated immunity in chickens [10]. Therefore, the S and N proteins, in parallel development, are widely used as antigen targets for IBV detection [11,12].

Monoclonal antibodies (mAbs), with the characteristic of high specificity, simple purification, and stable sources, are broadly utilized in the field and research as a reliably powerful tool for grouping and differentiating virus serotypes. The antigenic characterization of IBV strains by using mAbs directed against different epitopes on the S, N and M proteins could provide valuable information regarding antigenic relationships and variations [13,14,15]. Moreover, mAbs specific to Taiwan IBV strains have been used in a serum blocking ELISA, providing a simple and rapid method for detecting the wild-type IBV infection [16]. In addition, nucleic acid tests such as RT-PCR and other modifications of ELISA have been commonly used to identify strains of IBV in the field [4,17,18,19,20]. However, these methods are costly and time consuming, requiring skilled technicians to perform respective procedures in an equipped laboratory.

The immunochromatographic strip (ICS) test, or lateral flow assay, is an antibody-based technique, referring to the migration of antigen-antibody complexes through a filter matrix such as nitrocellulose strips [21,22]. During the test, gold nanoparticles-conjugated antibodies bind to the antigen of interest, and the complexes are then immobilized in the support matrix by unlabeled antibodies bound to the matrix. ICSs are widely designed for use at point-of-care as they provide cheap, simple and rapid tests desirable in many industries [23,24,25,26]. The objective of this study is to develop an ICS test for on-site antigen detection of IBV based on virus-specific mAbs. The use of the detection strip was validated by samples collected from experimental infections. This rapid IBV test is anticipated to benefit disease surveillance and control.

## 2. Results

### 2.1. Characteristics of mAbs against IBV

**Figure 1:**
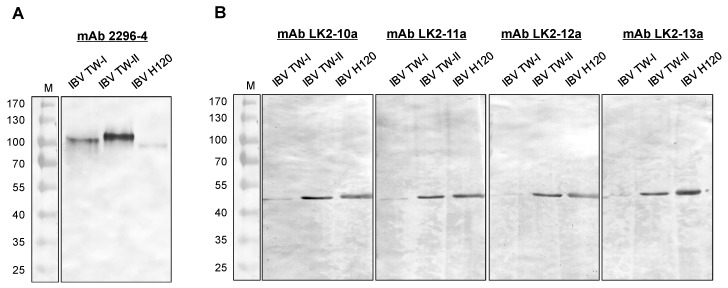
Characterization of monoclonal antibodies. Western blot was performed to verify the binding specificity of the monoclonal antibody (mAb) 2296-4, (**A**) LK2-10a, LK-11a, LK2-12a and LK-13a; (**B**) to the IBV TW-I, TW-II and H120.

A total of five IBV-specific mAbs (2296-4, LK2-10a, LK2-11a, LK2-12a and LK2-13a) were obtained through a series of subclonings and screenings in this study. The cross-reactivity of mAbs against IBV strains was assessed through Western blot. Of the five mAbs, 2296-4 was observed to interact with the spike protein subunit (approximately 90 kDa) of IBVs, including two locally dominant genotypes (TW-I and TW-II) and the Mass type H120 (Figure 1A). Four other mAbs recognized the N protein (approximately 45 kDa) of the three IBVs (mAbs LK2-11a and LK2-12a depicted in Figure 1B). None of them reacted to the allantoic fluid (AF) from uninfected specfic-pathgen free (SPF) chicken embryos and other avian respiratory viruses, including the avian influenza virus (AIV), Newcastle disease virus (NDV) and infectious laryngotracheitis virus (ILTV).

### 2.2. Analysis of the Tracer (mAb-Gold Conjugate)

**Figure 2:**
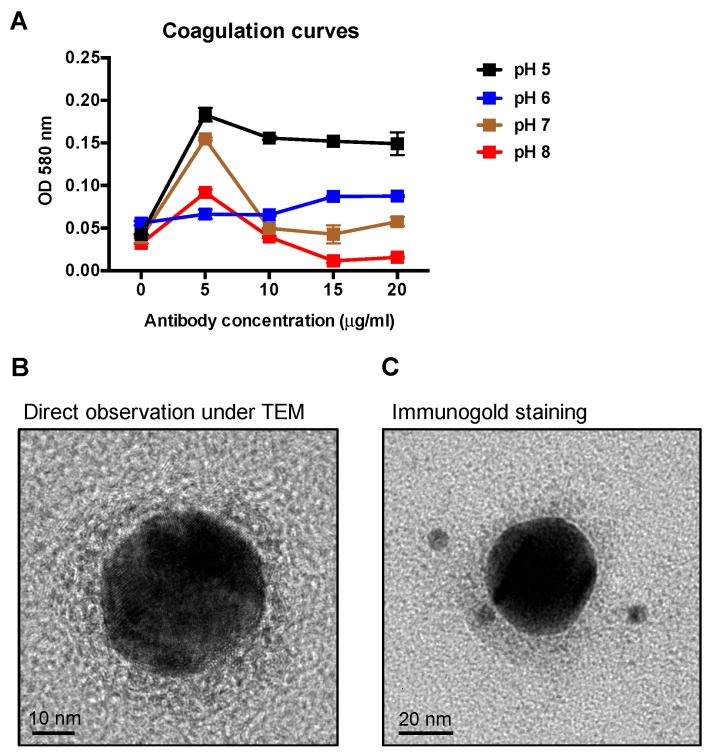
Coagulation curve analysis and immunogold staining of colloidal gold-labelled mAb. (**A**) The optimal pH value of gold particles and the concentration of mAb LK2-11a for stabilizing colloidal gold particles were determined by the coagulation curve analysis. The OD absorbance of each sample was determined at 580 nm. After the tracer (the mAb-gold complexes) was prepared, it was examined using transmission electron microscopy; (**B**) and stained by the goat anti-mouse IgG 6-nm gold conjugate (**C**).

The optimal pH value of gold particles and the concentration of mAbs for the adsorption of colloidal gold conjugation were determined by coagulation curves. The colloidal gold solution was adjusted to four different pH values between five and eight, physiological conditions for antibodies. Different concentrations of mAbs were added to each colloidal gold solution. Each point represented the absorbance at OD 580 nm after an additional 10% NaCl was added. The lower OD of the mAb-gold conjugate indicates the dispersed gold nanoparticle distribution in the solution as small particles have higher light transmittance, thereby indicating that the antibodies sufficiently stabilize the gold nanoparticles in the given pH environment. As indicated in Figure 2A, the lowest OD value corresponded with a mAb concentration of 15 μg/mL and a colloidal gold solution at pH 8. After the tracer was prepared, transmission electron microscopy (TEM) and immunogold staining were performed to examine the binding of the mAb with gold nanoparticles. As seen in Figure 2B, the 40-nm gold particle was surrounded by a grey layer, which was confirmed to be the mAbs through immunogold staining (Figure 2C).

### 2.3. Assembly of the ICS and Detection of IBVs and Other Avian Respiratory Viruses

**Figure 3:**
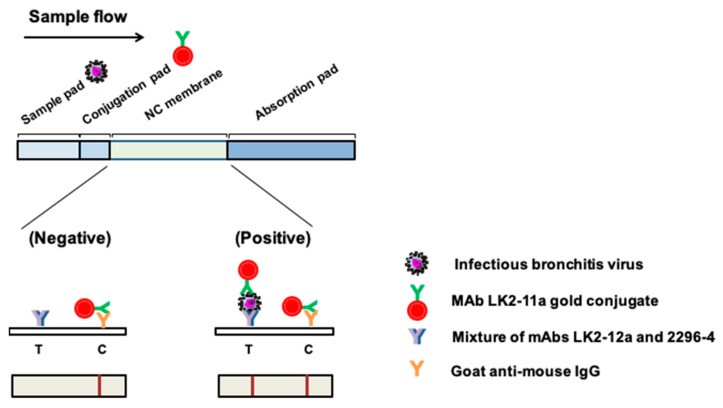
Principle of operation for the immunochromatographic strip. The mAb LK-11a-gold conjugate was placed on the conjugation pad as a tracer (detection antibody). A mixture of mAbs LK2-12a and 2296-4 served as the capture antibody on the test line (**T**). Goat anti-mouse IgG served as the control on the control line (**C**).

**Figure 4:**
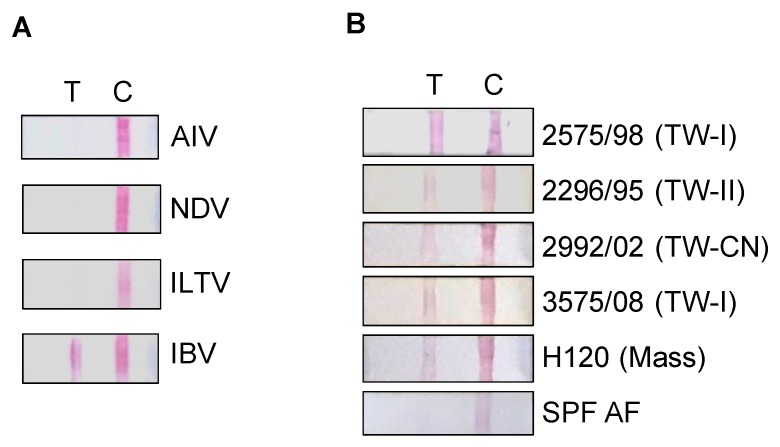
Avian infectious bronchitis virus (IBV) detection by the immunochromatographic strip (ICS). (**A**) Allantoic fluid (AF) of avian respiratory pathogens, avian influenza virus (AIV), Newcastle disease virus (NDV), infectious laryngotracheitis virus (ILTV) and infectious bronchitis virus (IBV) were tested with the ICS; (**B**) various IBV strains including 2575/98 (TW-I), 2296/95 (TW-II), 2992/02 (TW-China variant), 3575/08 (TW-I), H120 (Mass), and AF from specific-pathogen-free (SPF) chicken embryos were examined using the ICS. T: Test line; C: Control line.

The schematic illustration of ICS is indicated in Figure 3. Various samples of avian respiratory pathogens, including the AF (10^5.5^ EID_50_) of AIV, NDV, ILTV and IBV, were used to test the specificity of the assembled ICS. The test line was found to respond to IBV only but not to other respiratory diseases’ antigens (Figure 4A). The ICS indicated high specificity in IBV antigen detection. In addition, the cross-reactive detection against different strains of the IBV provided by this ICS test was evaluated. Results demonstrated that the ICS was capable of detecting numerous IBV genotypes, including TW-I, TW-II, Mass, and the TW-China variant, but not the non-infectious AF (Figure 4B). Morover, the detection limit against the IBVs was evaluated. As indicated in Table 1, IBV 2575/98 (TW-I) exhibited the best detection limit at 10^4.4^ EID_50_, followed by H120 (Mass type) at 10^4.6^ EID_50_, 2992/02 (TW-CN variant) at 10^4.8^ EID_50_ and IBV 2296/95 (TW-II) at 10^4.8^ EID_50_.

### 2.4. Validation of ICS in Clinical Settings

To validate the use of ICS in clinical settings, a total of 18 throat and cloacal swab samples were collected from IBV 2992/02-infected chickens at one, three and five days post-infection. RT-PCR was used simultaneously as a reference test. Results indicated that ICS successfully detected viral antigens from throat swabs sampled on one (3/3), three (2/3) and five (3/3) days post-infection (dpi), with highly consistent results from RT-PCR (Table 2). For cloacal swabs, viral antigen was not detected until 5 dpi through RT-PCR, in agreement with the findings of the ICS detection. Collectively, the ICS is suitable for early viral detection and reveals sensitivity comparable to that of RT-PCR.

## 3. Discussion

Many highly contagious viral diseases can affect the respiratory system of poultry, such as Newcastle disease, avian influenza, infectious laryngotracheitis and infectious bronchitis. Making a differential diagnosis of these diseases in the field is difficult, because affected chickens exhibit similar clinical signs: Respiratory distress and watery discharge from eyes and nostrils. Therefore, the rapid and accurate diagnosis of the disease facilitates disease management. Avian infectious bronchitis has posed a threat in the poultry industry worldwide. Even though protection schemes have been applied by means of a live attenuated IBV vaccine, low levels of cross-protection have been observed, [8,27]. In such a case, a prompt identification of IBV would be highly beneficial.

In this study, we successfully developed an ICS rapid test, which can be completed within 10 min to identify the IBV infection. To the best of our knowledge, this is the first report of IBV antigen detection by using an ICS. The results of comparing the reference RT-PCR test with our ICS implied that the ICS could detect antigens as early as 1 dpi from throat swab samples and 5 dpi from cloacal swab samples. The detection limit of the ICS strip was approximately 10^4.5^ to 10^4.8^ EID_50_. The results of our study were slightly better than those reported for the liquid-phase blocking ELISA in Lougovskaia et al. [28] with the detection limit of the H120 strain reported to be 10^5.8^ EID_50_. The nature of mAbs developed in this study is specific to the IBV among avian respiratory pathogens. Furthermore, by employing broadly-reactive mAbs of IBV, this described ICS displayed cross-reactivity against multiple types of IBVs, including two local Taiwan genotypes, recombinant variants, and the Mass type strain. Additional studies should be taken to ensure an antigen–antibody reaction against some other common strains worldwide; for example, Arkansas, Conn, JMK or QX-IBV.

Based on the five mAbs we have obtained, the performance serving the capture antibody or detection antibody among several pairs of the mAbs were examined. Several different tracers were produced as described in the methods section. Various 1:1 combinations of the mAb mixture were also prepared, consisting of one mixture of both anti-N mAbs and one of anti-N and anti-S mAbs, with a total of 17 combinations tested. Both the tracers and test line mixtures were applied to the ICS. A positive IBV TW-I type AF was used as an antigen, and SPF AF was used as a negative control to determine the detection capacity of each combination. It was found that the use of mAb LK-10a and LK2-13a in the ICS generated non-specific signals and thus was ruled out as an option. In the preparation of the tracer, the stability of the mAb-gold conjugate is critical for optimizing the ICS strip’s performance. In this study, an mAb concentration of 15 μg/mL with colloidal gold solution at pH 8 exhibited the most stable condition in coagulation curves. As depicted in TEM images, the tracers were thoroughly dispersed in the solution, showing a unimodal particle distribution with an average size of 40 nm.

In summary, the ICS developed in this study was a rapid and simple method for detecting IBV antigens. Moreover, mAbs employed on the strip and the colloidal gold-mAb tracer were ideal for identifying various IBV genotypes. The ICS has the potential to be employed as a screening tool for diagnosing IBV infections in the poultry industry. Further studies evaluating the sensitivity and specificity of ICS detection using the field samples are warranted.

## 4. Materials and Methods

### 4.1. Ethics Statement

The care and use of experimental animals were approved by the Institute Animal Care and Use Committee, National Taiwan University (approval no. NTU-103-EL-3 issued on 1 March 2014). All animal experiments were conducted in accordance with approved guidelines.

### 4.2. Virus Propagation and Titration

IBV strains 2575/98 (TW-I), 2296/95 (TW-II), 3575/08 (TW-I), 2992/02 (TW-China variant), and H120 (Mass) were propagated in 10-day-old SPF chicken embryos through the allantoic route as previously described [29]. AIV A/chicken/Taiwan/2838V/00 [30], NDV strain TW-2/00 [31], and a clinical isolate of ILTV were also propagated. The virus titers of IBVs were determined using the method of Reed and Muench [32] in SPF chicken embryos and were expressed as the 50% embryo-infective dose (EID_50_). For viral antigens used in Western blots, the AF containing the virus was further concentrated and sucrose-gradient-purified as previously described [16].

### 4.3. Production of mAbs

Female BALB/c mice (Lasco, Taipei, Taiwan) were intraperitoneally immunized with 1 mL of IBV 2575/98 (10^7.4^ EID_50_/mL), and hybridoma cells were produced as previously described [16]. Positive hybridomas were screened through Western blot analysis against IBV antigens (as subsequently described). After two subclonings by limiting dilution, desired clones, which recognized the S or N proteins of IBV, were inoculated intraperitoneally into incomplete Freund’s adjuvant (Sigma, St. Louis, MO, USA)-primed BALB/c mice to produce ascites containing mAbs. The mAbs were affinity purified using the Protein A IgG purification kit (Pierce Biotechnology, Rockford, IL, USA) according to the manufacturer’s instructions.

### 4.4. Western Blot Analysis

One μg of IBV 2575/98 (TW-I), 2296/95 (TW-II), and H120 viral antigens were mixed with an equal volume of SDS-PAGE sample buffer (100 mM Tris-HCl, 200 mM DTT, 4% SDS, 0.02% bromophenol blue, 20% glycerol, pH 6.8) and boiled for 5 min. The protein solution was separated on 10% SDS-PAGE. Protein gels were transferred onto a 0.45-μm nitrocellulose membrane (Bio-Rad, Richmond, CA, USA). After the transfer, the membrane was soaked in blocking buffer (5% skim milk in PBS) at room temperature for 1 h, and the hybridoma supernatant diluted 1:5 in blocking buffer was then added. After three washes, the membrane was incubated with peroxidase-conjugated goat anti-mouse IgG (H+L) (Jackson ImmunoResearch, West Grove, PA. USA) diluted 1:2000 in blocking buffer at room temperature for 1 h. Following three further washes, protein blots were detected using a TMB membrane peroxidase substrate (KPL, Gaithersburg, MD, USA).

### 4.5. Coagulation Curves

Coagulation curves were analyzed as previously described [33,34]. Briefly, 100 μL of purified mAbs in various concentrations (0, 5, 10, 15 and 20 μg/mL) was added to 500 μL of the colloidal gold nanoparticle solution (40 nm, Sigma, St. Louis, MO, USA) with pH values varying from five to eight. After 1 min, an additional 100 μL of 10% NaCl solution was mixed and incubated for 5 min. The OD absorbance of each sample was determined at 580 nm by using an automated reader (Multiskan FC Microplate Photometer, Thermo, Waltham, MA, USA) with a blank control consisting of 500 μL of colloidal gold and 200 μL of H_2_O mixture. OD absorptions at each pH and mAb concentration were obtained in triplicate. The optimal pH value of gold particles and the concentration of mAbs for stabilizing colloidal gold particles were determined.

### 4.6. Conjugation of mAbs with Colloidal Gold

The mAb-colloidal gold conjugate (tracer) was prepared as previously described [35] with modifications, and the pH value of colloidal gold and the concentration of mAb used during conjugation were optimized from coagulation curves described above. Briefly, 20 mL of colloidal gold was adjusted to pH 8.0. Furthermore, 330 μL of the purified mAb LK2-11a was added and mixed vigorously for 30 min at room temperature, followed by an additional 30 min of stirring with 5% (*w*/*v*) bovine serum albumin (BSA) to block the excess reactivity of colloidal gold. After centrifugation at 4500× *g* for 45 min, the mAb-colloidal gold pellet was washed with 2 mM borax buffer containing 0.1% PEG-20000 (pH 9.0) twice and then resuspended in 1 mL of 2 mM borax buffer containing 0.1% PEG-20000 and 10% sucrose (pH 9.0).

### 4.7. Immunogold Staining

For immunogold labelling of the mAb tracer, eight μL of tracer sample was deposited onto a glow-discharged carbon-coated grid for 2 min and fixed with 4% paraformaldehyde for 5 min. Following PBS washing, the grid was blocked with 1% (*w*/*v*) BSA for 1 h. The grid was then stained with 6-nm gold-conjugated goat anti-mouse IgG (Jackson ImmunoResearch) diluted in blocking solution (1:20) for 1 h and washed three times in PBS. Particles were visualized under a 200-kV high-resolution transmission electron microscope (FEI Tecnai TF20, Hillsboro, OR, USA).

### 4.8. Praparation and Operation of ICS for IBV Detection

The ICS consisted of four main materials as indicated in Figure 3: A sample pad, conjugate pad, nitrocellulose membrane and absorption pad (Prisma Biotech Corporation, Taipei, Taiwan). The sample pad and conjugate pad were first treated with 20 mM phosphate buffer containing 1% BSA, 0.5% tween 20, 0.05% sodium azide and 5% sucrose at pH 7.4; they were subsequently dried at 37 °C. Then, two μL of the mAb LK2-11a gold conjugate (tracer) generated in this study was used on the conjugation pad. The mixture of two mAbs, LK2-12a (0.5 μg) and 2296-4 (0.5 μg) and 0.6 μg of the goat anti-mouse IgG (H+L) (Jackson ImmunoResearch) were loaded onto the NC membrane—serving as a test line and control line, respectively—by means of an XYZ 3050 dispensing platform (BioDot, Irvine, CA) equipped with the BioJet Quanti 3000 dispensers. Upon use, the strip was inserted into the sample solution tube, ensuring the sample pad of ICS was fully reacted with the sample. The results were interpreted within 10 min.

### 4.9. Experimental Infection in Chickens

Two-week-old SPF chickens (JD SPF Biotech, Miaoli, Taiwan) were intranasally infected with IBV 2992/02 [5] at a dose of 10^6^ EID_50_ per chicken. Throat and cloacal swabs were collected in tryptose phosphate broth (Difco Labs, Detroit, MI) at one, three and five dpi. The swabs were clarified by centrifuging at 3000× *g* for 20 min and stored at −80 °C until further testing with ICS and RT-PCR.

### 4.10. RT-PCR

Viral RNA was extracted from swab samples by using the QIAamp Viral RNA kit (Qiagen, Valencia, CA) as per the manufacturer’s instructions. Subsequently, a 25 μL reaction was set up consisting of 2.5 μL of template RNA, 0.5 μL of 2.5 mM dNTP, 2.5 μL of 10× buffer, 0.25 μL each of forward and reverse primers (50 μM) (Mission Biotech, Taipei, Taiwan) for targeting the N protein gene, 0.2 μL of RNase inhibitor (Invitrogen, Carlsbad, CA, USA), 0.2 μL of RealTaq DNA polymerase (Real Biotech, Taipei, Taiwan), 0.1 μL of MMLV (Invitrogen), and 18.5 μL of RNase-free water. Reverse transcription was performed at 40 °C for 30 min, followed by initial denaturation at 95 °C for 5 min. Then, 35 cycles were applied with 95 °C for 30 s, 55 °C for 30 s and 72 °C for 45 s. The final extension stage was performed at a temperature of 72 °C for 12 min and then maintained at 4 °C. PCR products were analyzed through the agarose gel electrophoresis.

## Figures and Tables

**Table 1:** Detection limit for various IBV strains by immunochromatographic strip.

IBV	Genotype	Detection Limit (EID_50_)
2575/98	TW-I	10^4.4^
2296/95	TW-II	10^4.8^
2992/02	Variant	10^4.8^
H120	Mass	10^4.6^

**Table 2:** Comparison of IBV detection by immunochromatographic strip and RT-PCR.

IBV Detection	Throat Swab			Cloacal Swab		
	1 dpi	3 dpi	5 dpi	1 dpi	3 dpi	5 dpi
ICS	3/3	2/3	3/3	0/3	0/3	1/3
RT-PCR	3/3	3/3	3/3	0/3	0/3	1/3
